# Supplementary figures and images for: Crystal structure and catalytic mechanism of PL35 family glycosaminoglycan lyases with an ultrabroad substrate spectrum
Source: eLife. 2025 May 19;13:RP102422. doi: 10.7554/eLife.102422 (PMC12088678; doi:10.7554/eLife.102422)

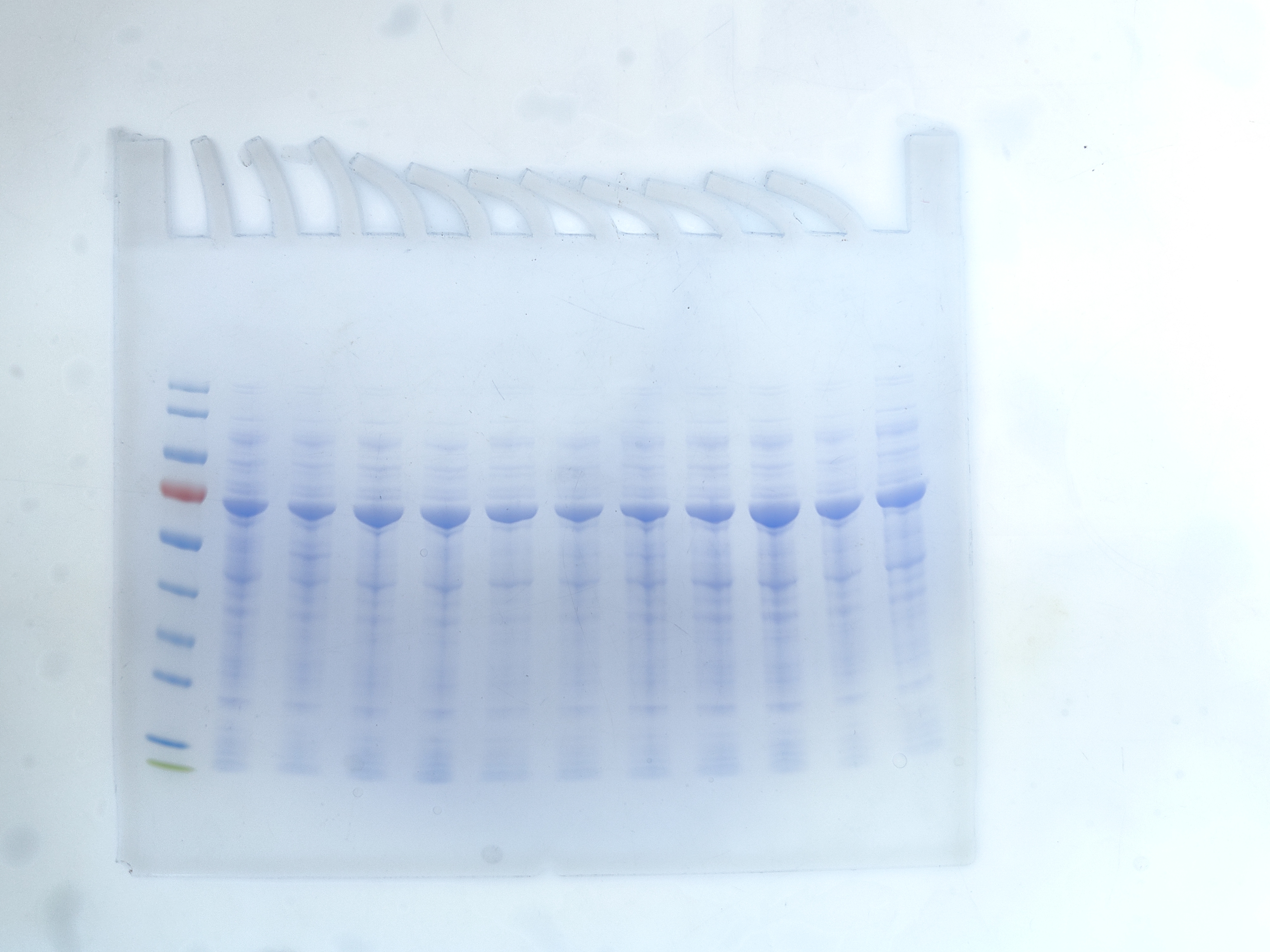

Supplement: Figure 4—figure supplement 2—source data 1. [file elife-102422-fig4-figsupp2-data1.zip › Figure 4-figure supplement 2 A-2.jpg]

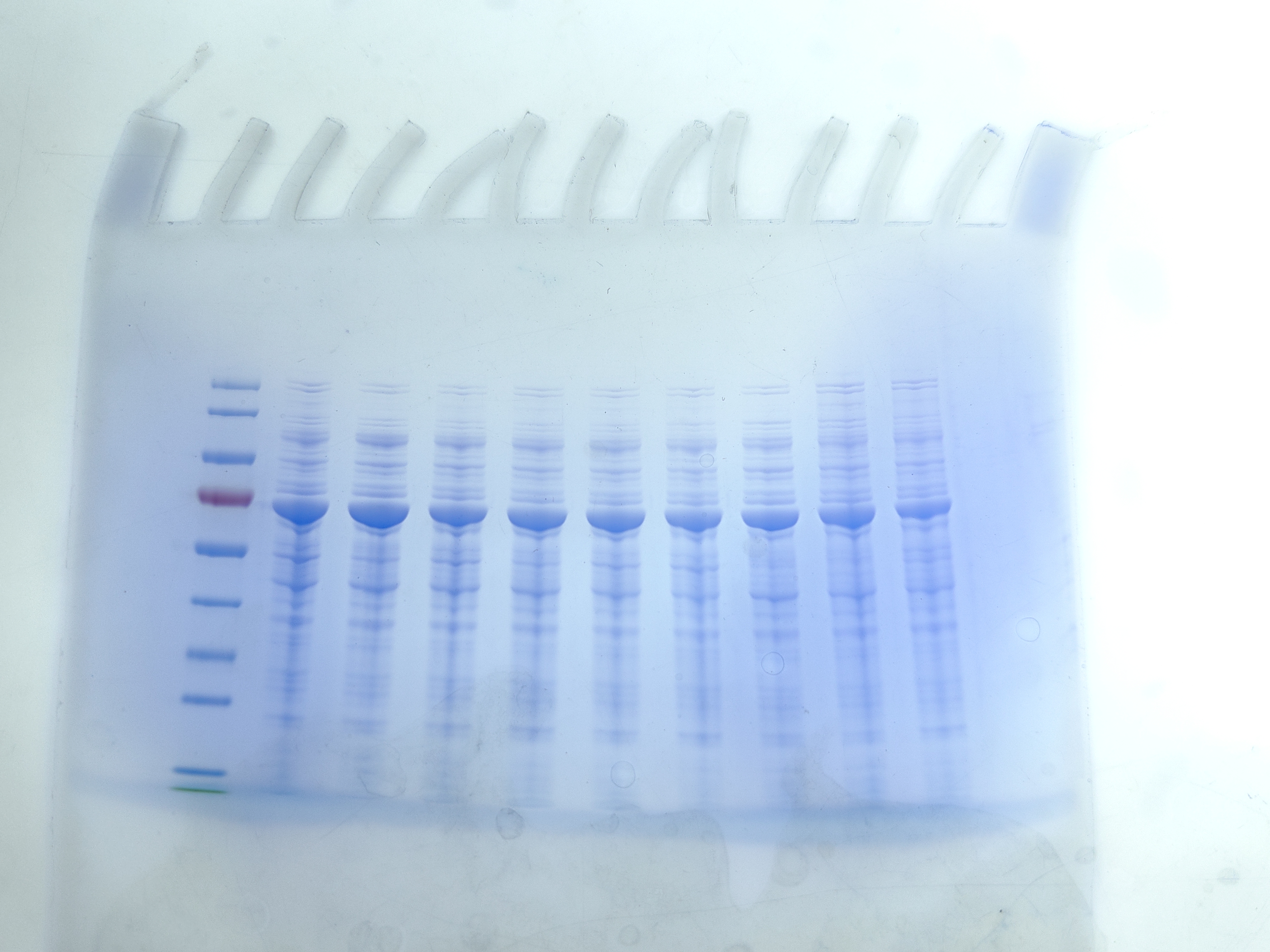

Supplement: Figure 4—figure supplement 2—source data 1. [file elife-102422-fig4-figsupp2-data1.zip › Figure 4-figure supplement 2 A-3.jpg]

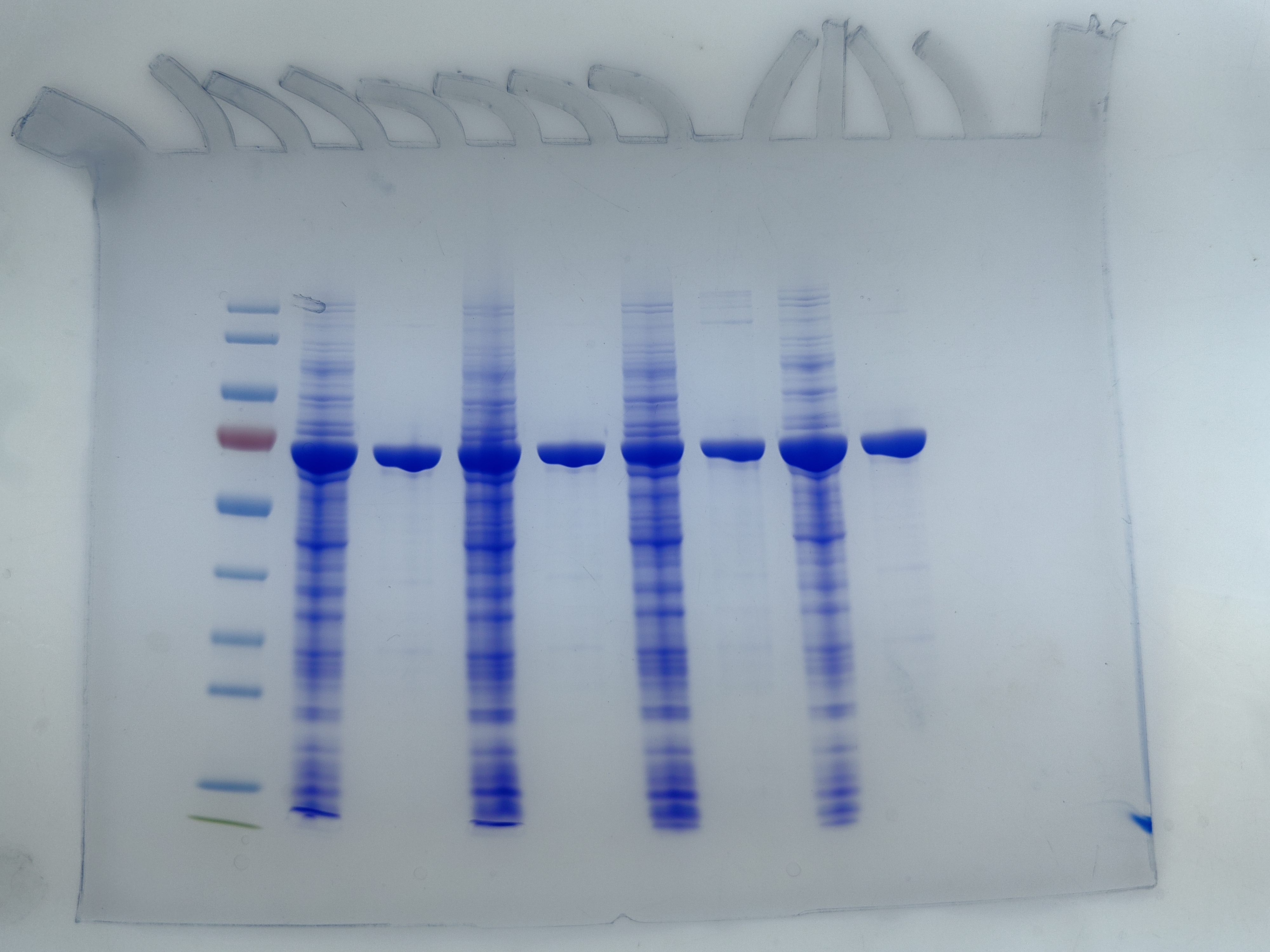

Supplement: Figure 4—figure supplement 2—source data 1. [file elife-102422-fig4-figsupp2-data1.zip › Figure 4-figure supplement 2 B-3.jpg]

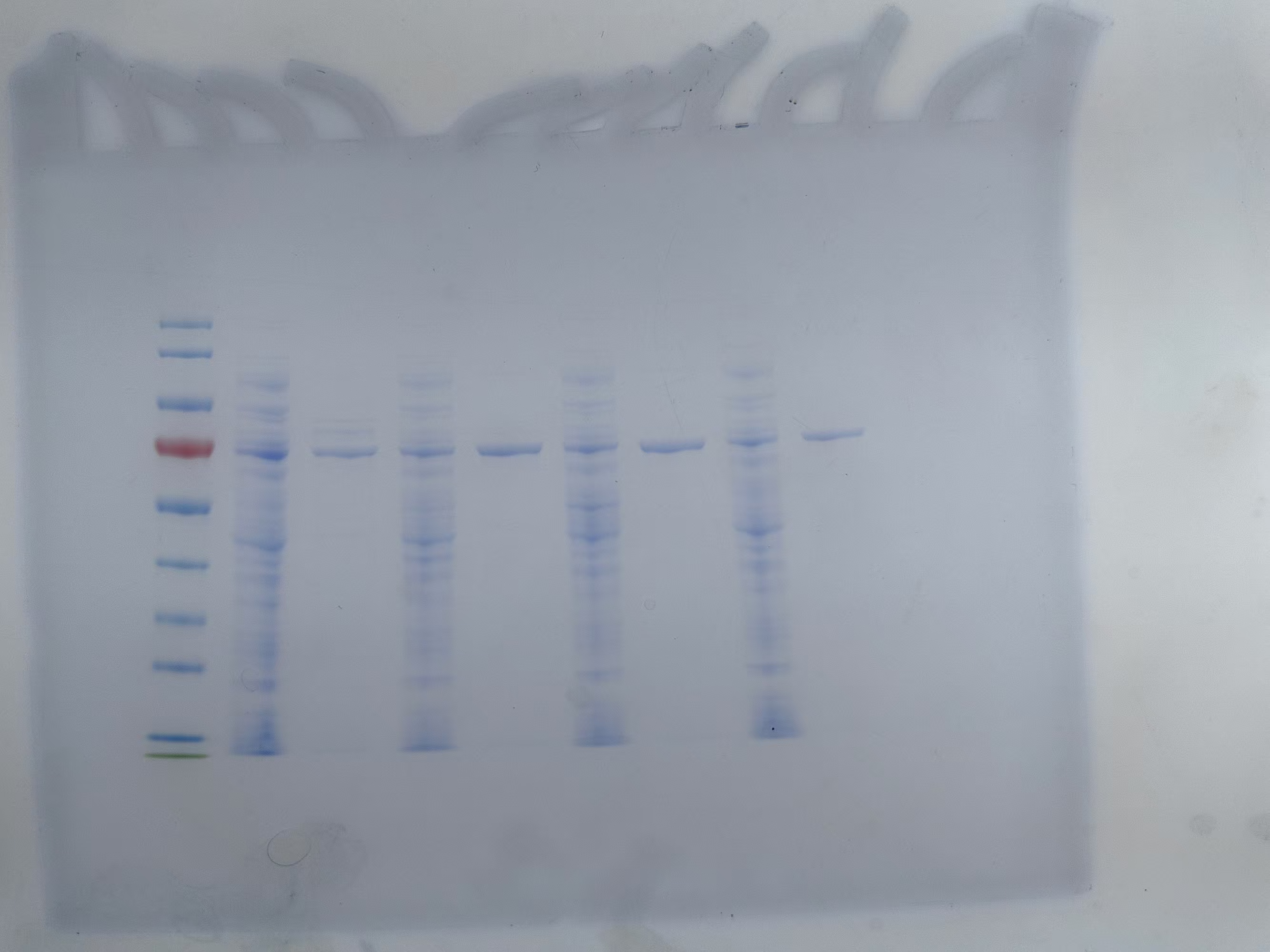

Supplement: Figure 4—figure supplement 2—source data 1. [file elife-102422-fig4-figsupp2-data1.zip › Figure 4-figure supplement 2 B-2.jpg]

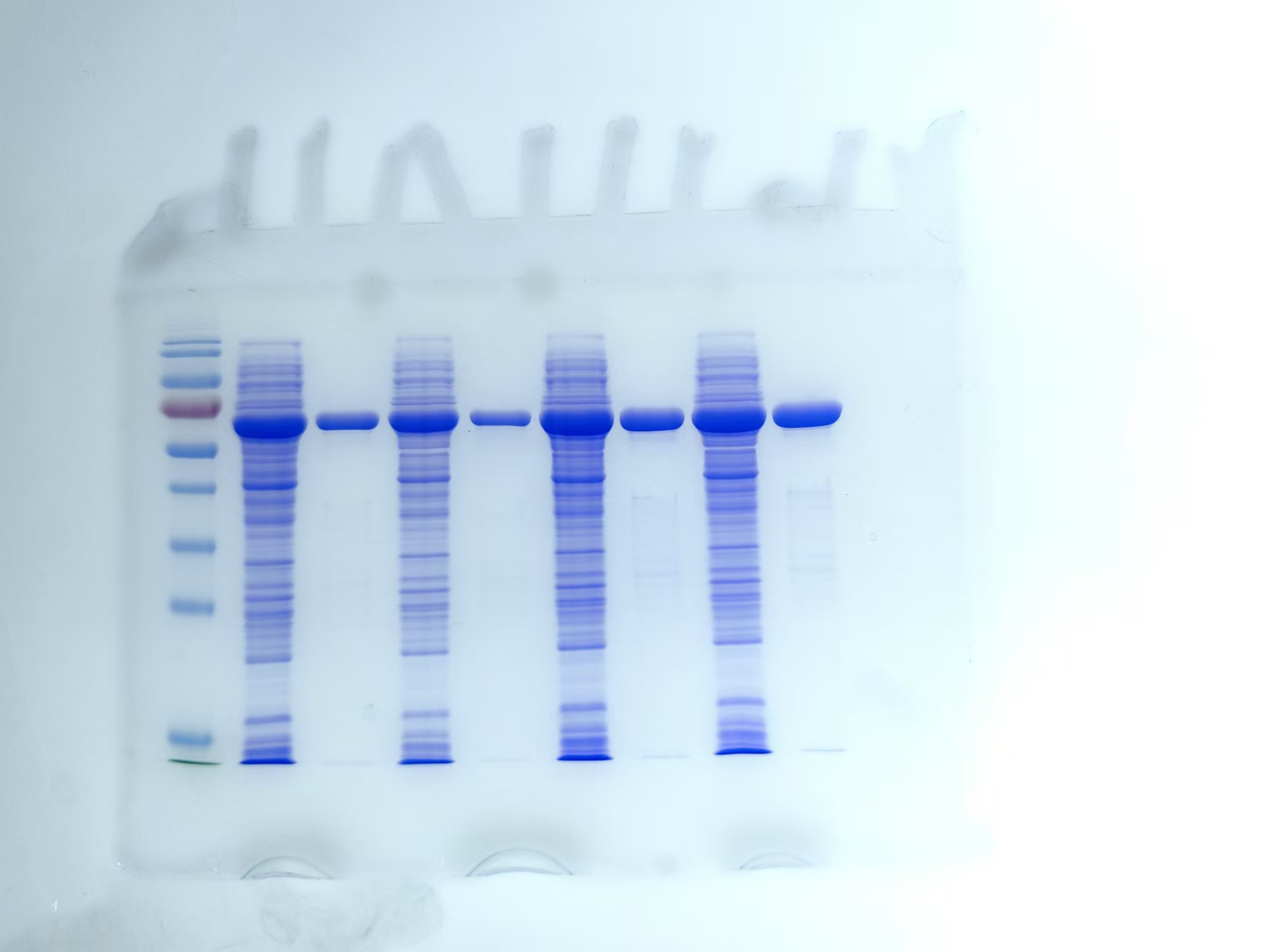

Supplement: Figure 4—figure supplement 2—source data 1. [file elife-102422-fig4-figsupp2-data1.zip › Figure 4-figure supplement 2 C-1.jpg]

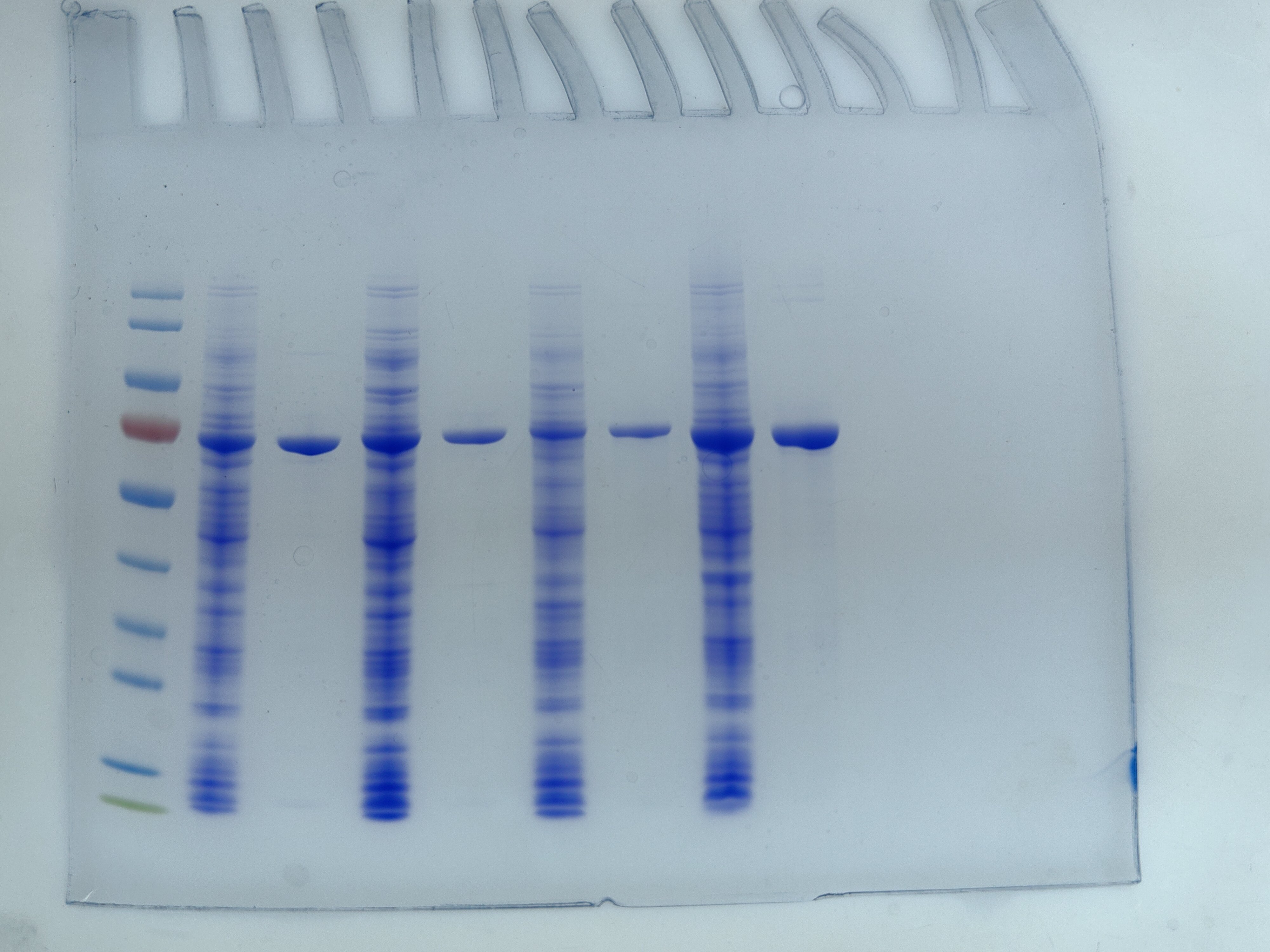

Supplement: Figure 4—figure supplement 2—source data 1. [file elife-102422-fig4-figsupp2-data1.zip › Figure 4-figure supplement 2 C-2.jpg]

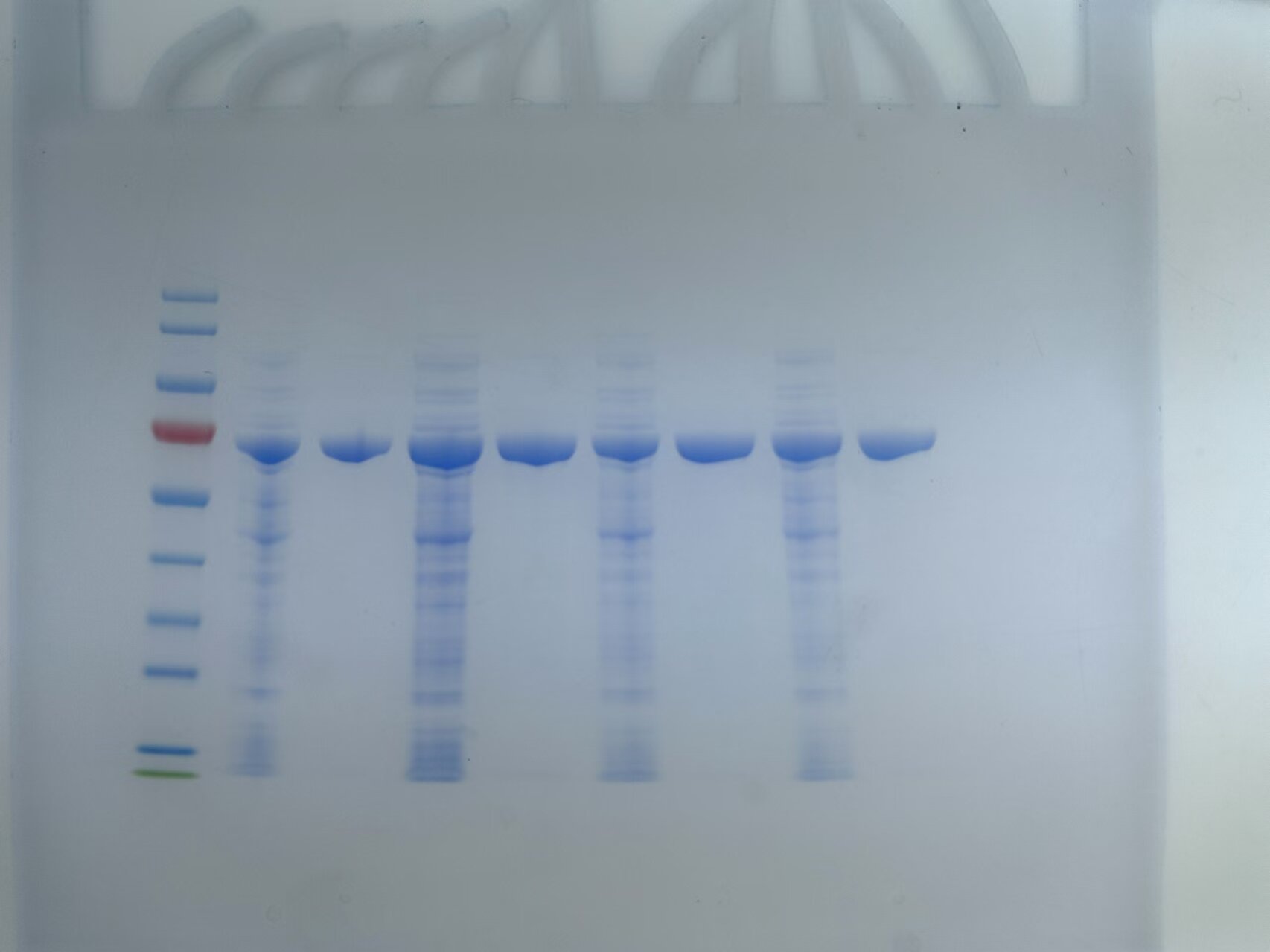

Supplement: Figure 4—figure supplement 2—source data 1. [file elife-102422-fig4-figsupp2-data1.zip › Figure 4-figure supplement 2 B-1.jpg]

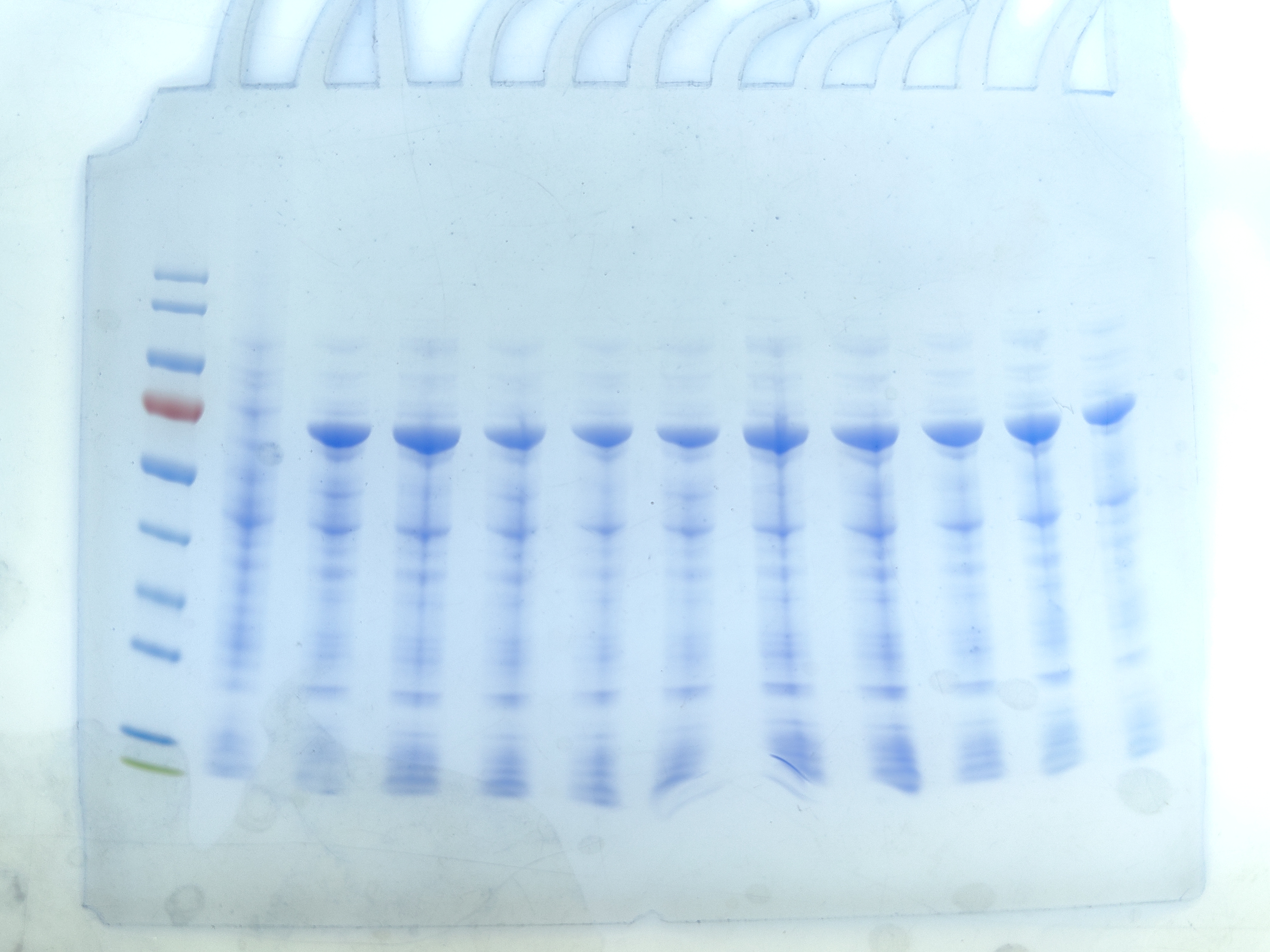

Supplement: Figure 4—figure supplement 2—source data 1. [file elife-102422-fig4-figsupp2-data1.zip › Figure 4-figure supplement 2 A-1.jpg]

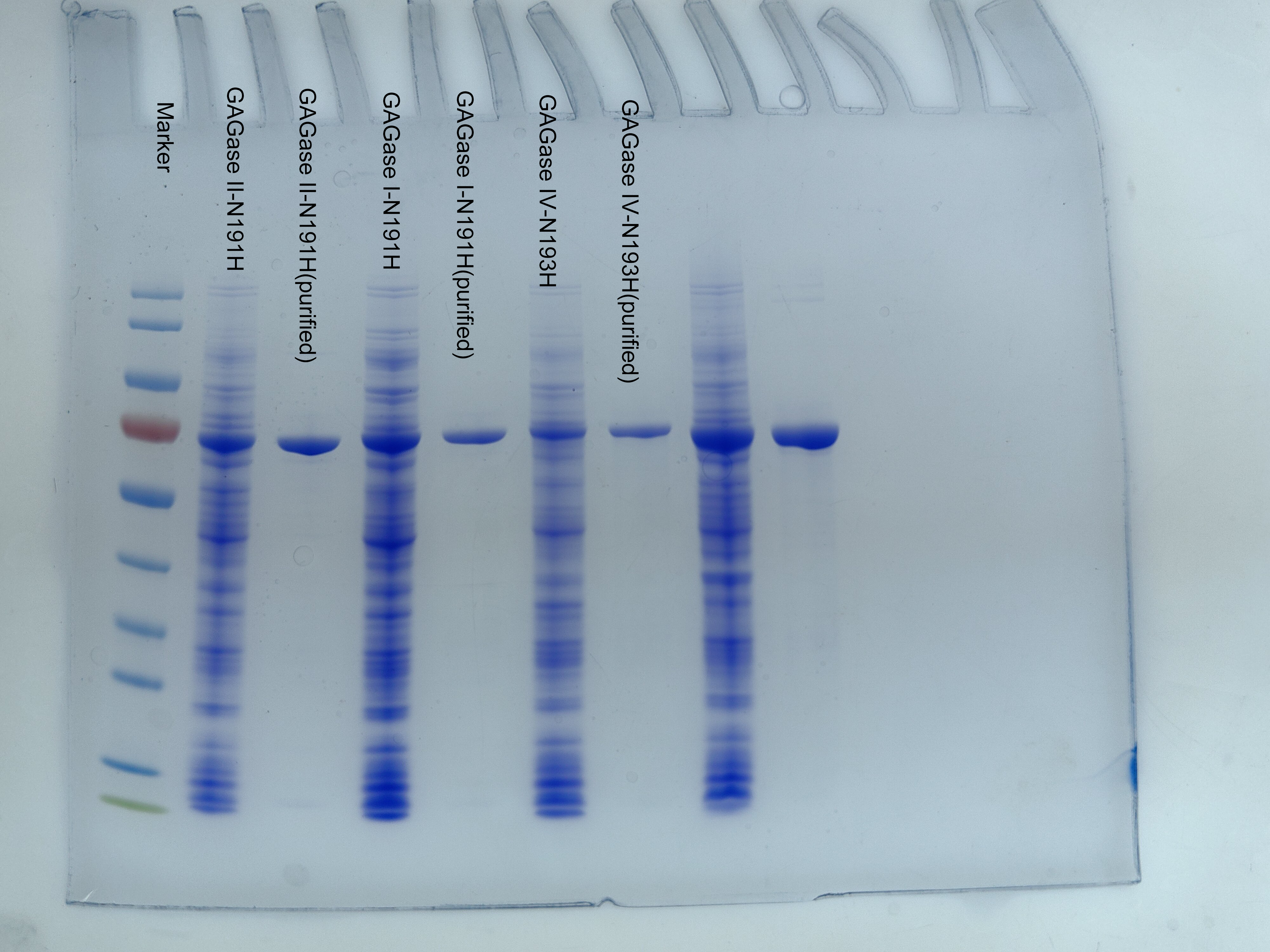

Supplement: Figure 4—figure supplement 2—source data 2. [file elife-102422-fig4-figsupp2-data2.zip › Figure 4-figure supplement 2 C-2.png]

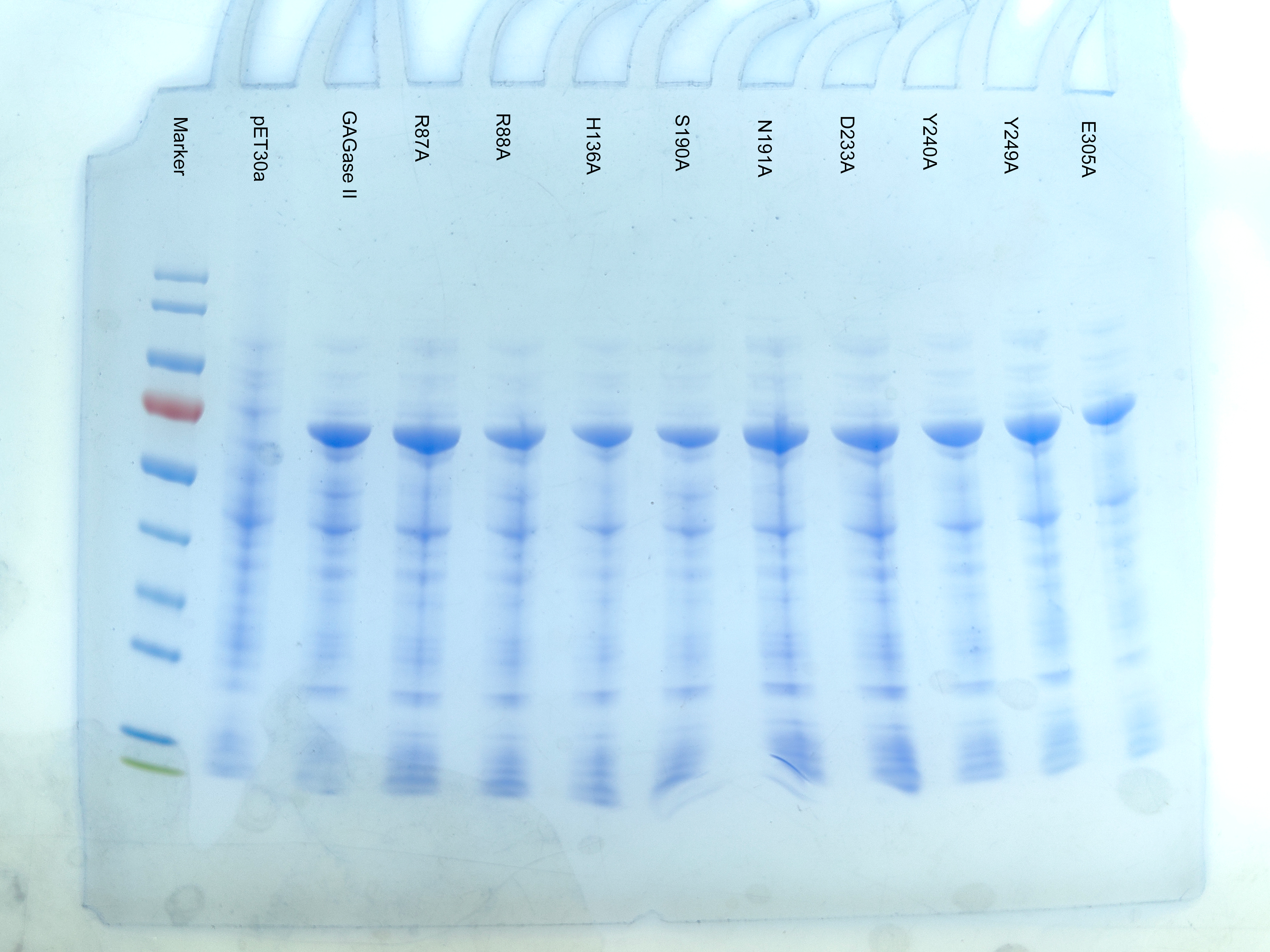

Supplement: Figure 4—figure supplement 2—source data 2. [file elife-102422-fig4-figsupp2-data2.zip › Figure 4-figure supplement 2 A-1.png]

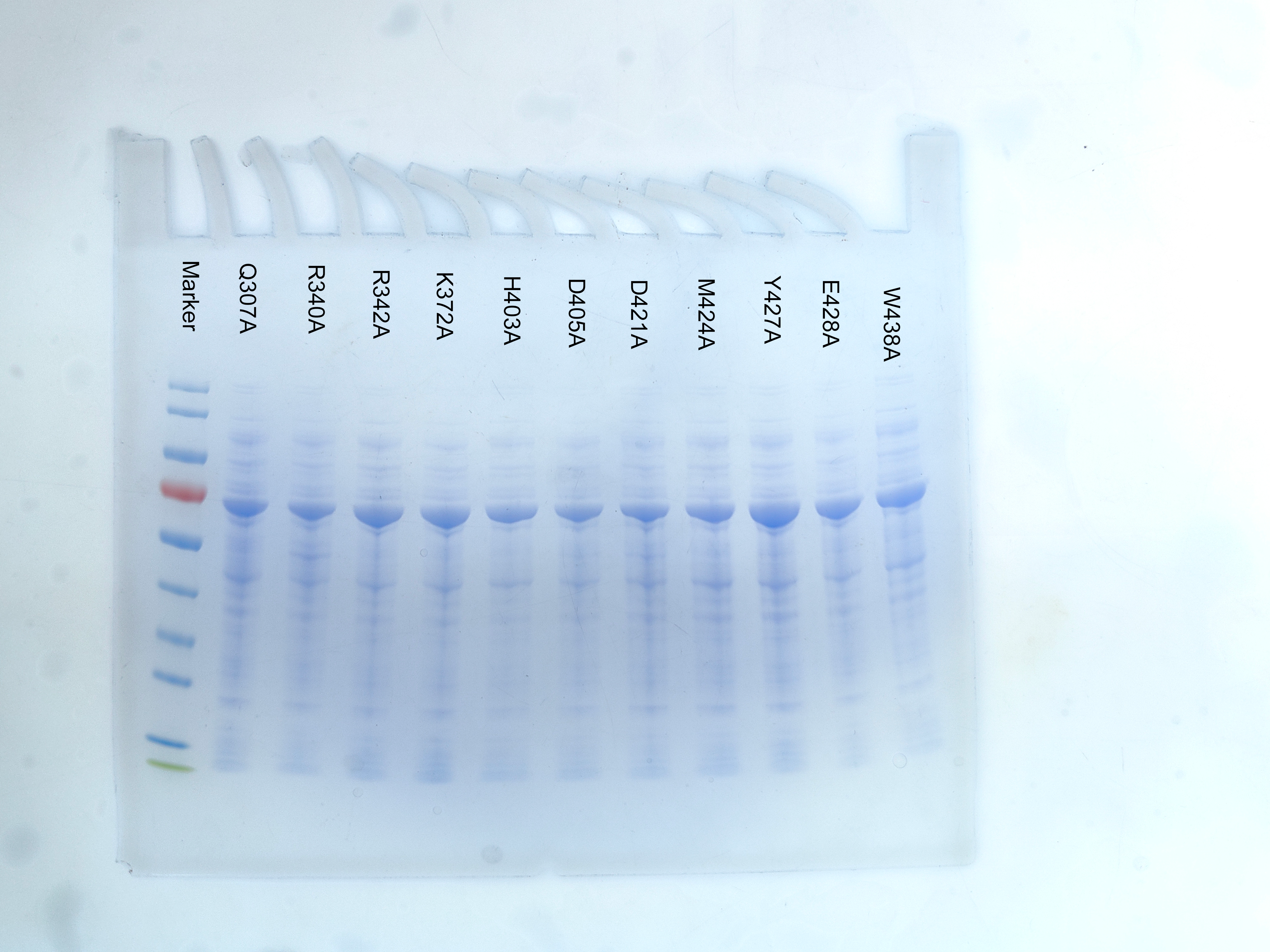

Supplement: Figure 4—figure supplement 2—source data 2. [file elife-102422-fig4-figsupp2-data2.zip › Figure 4-figure supplement 2 A-2.png]

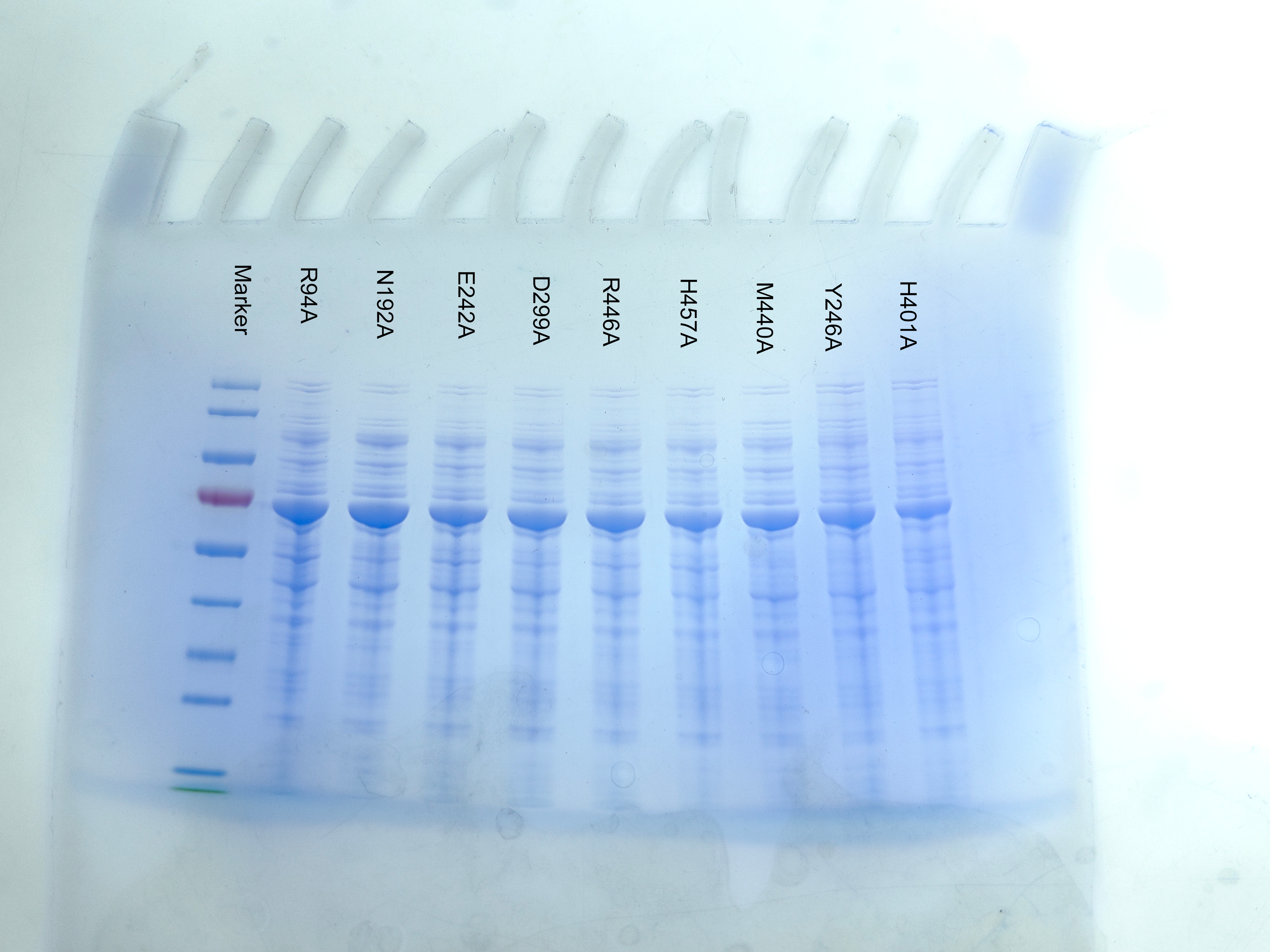

Supplement: Figure 4—figure supplement 2—source data 2. [file elife-102422-fig4-figsupp2-data2.zip › Figure 4-figure supplement 2 A-3.png]

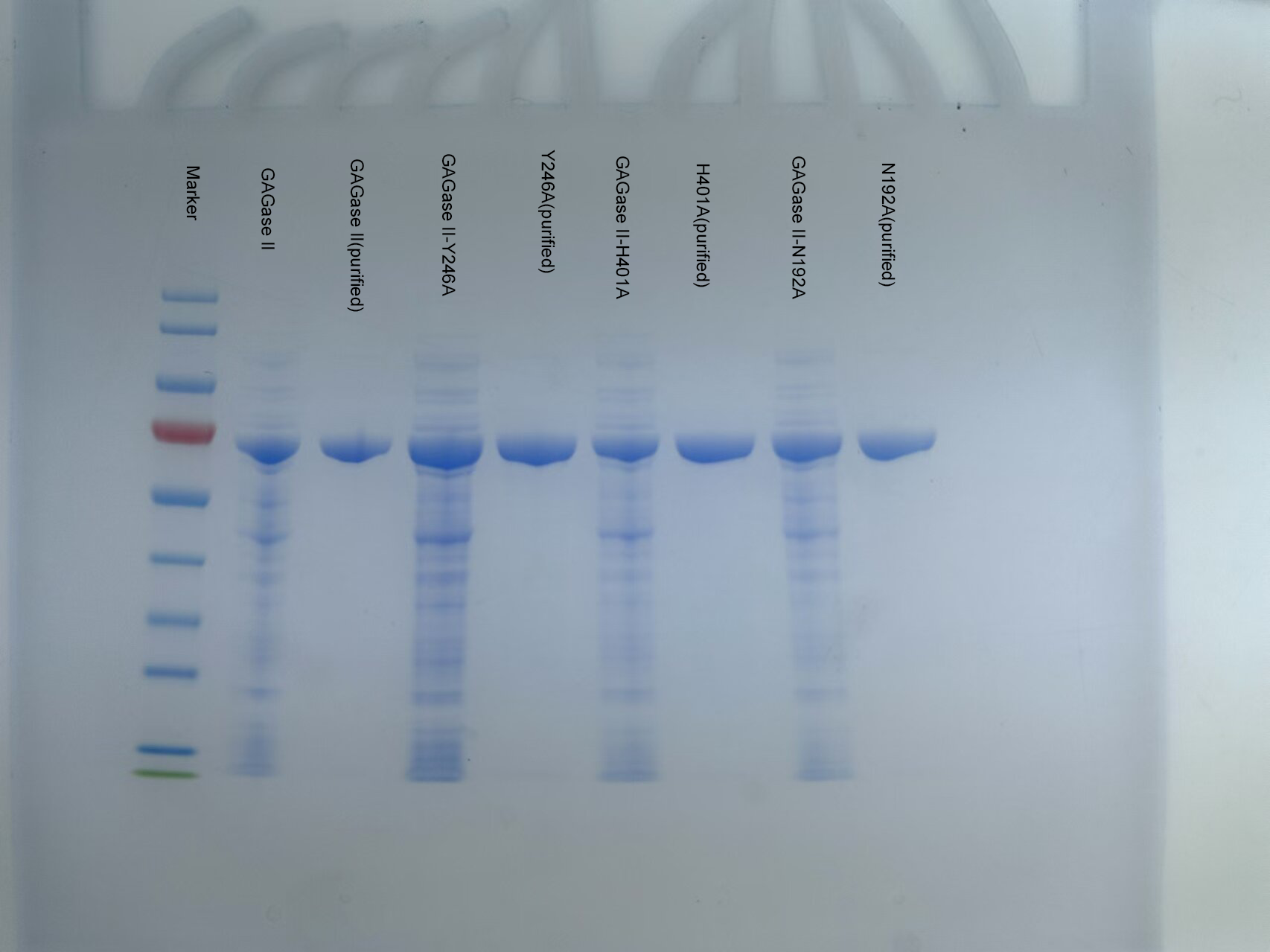

Supplement: Figure 4—figure supplement 2—source data 2. [file elife-102422-fig4-figsupp2-data2.zip › Figure 4-figure supplement 2 B-1.png]

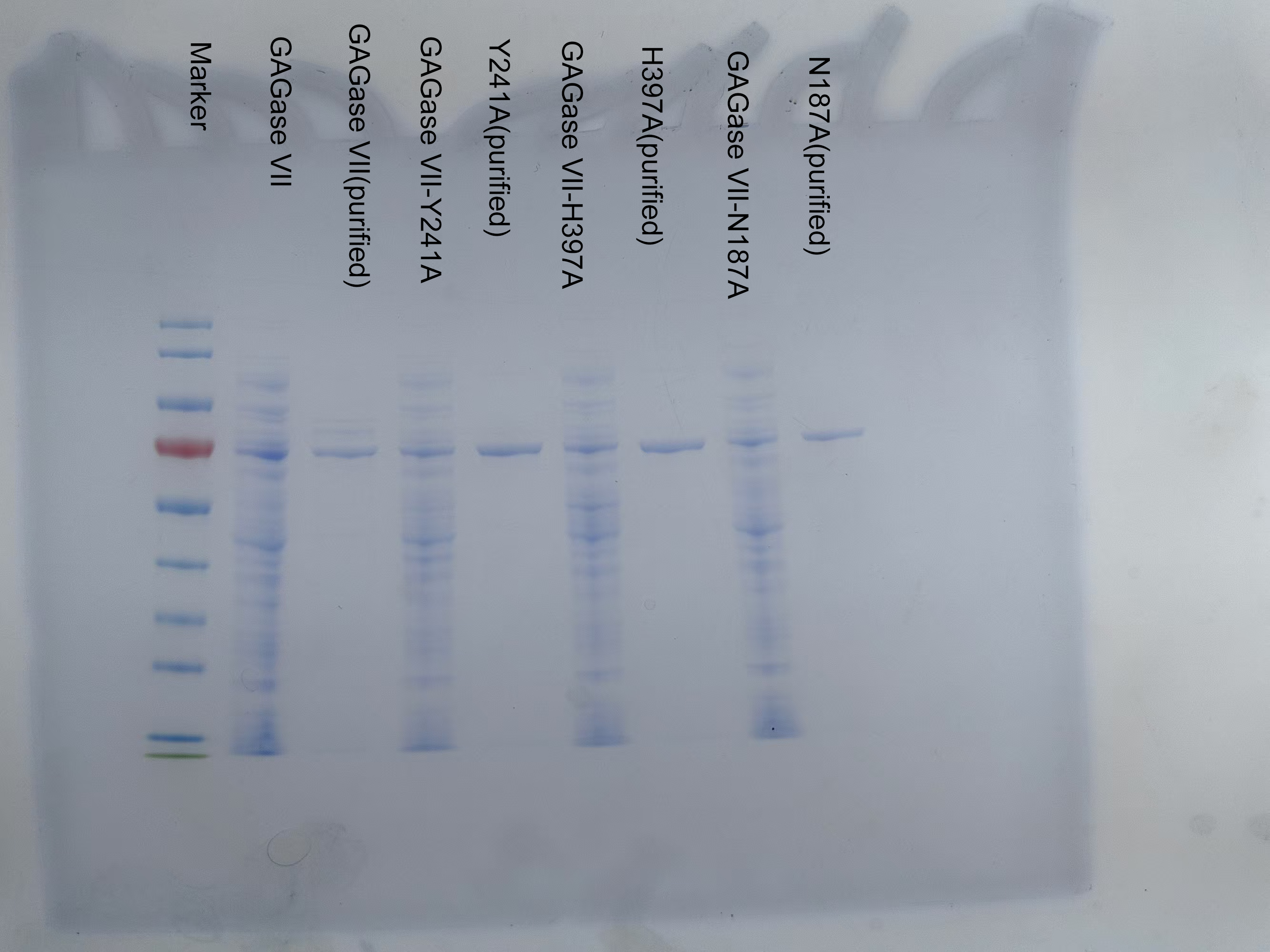

Supplement: Figure 4—figure supplement 2—source data 2. [file elife-102422-fig4-figsupp2-data2.zip › Figure 4-figure supplement 2 B-2.png]

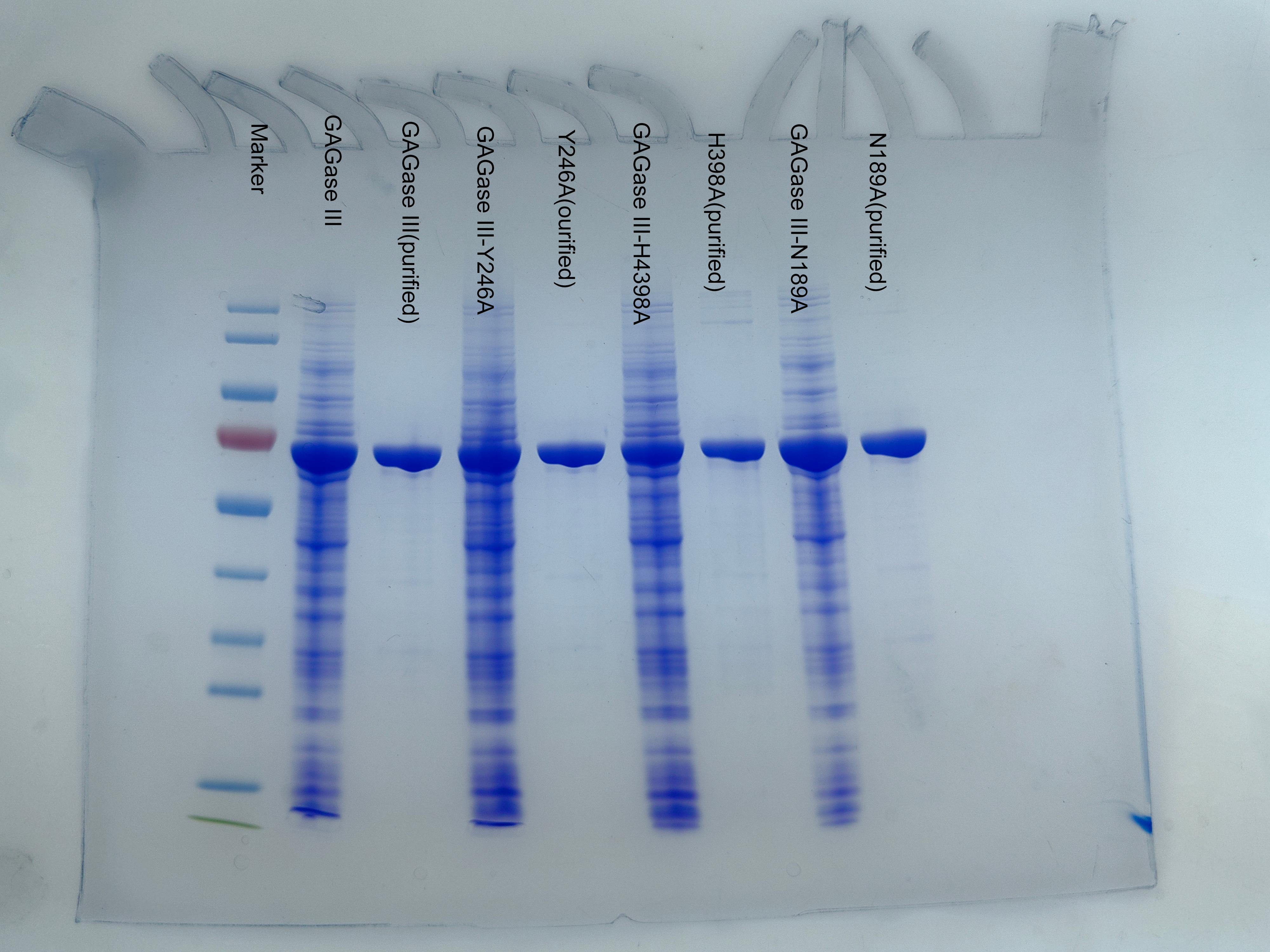

Supplement: Figure 4—figure supplement 2—source data 2. [file elife-102422-fig4-figsupp2-data2.zip › Figure 4-figure supplement 2 B-3.png]

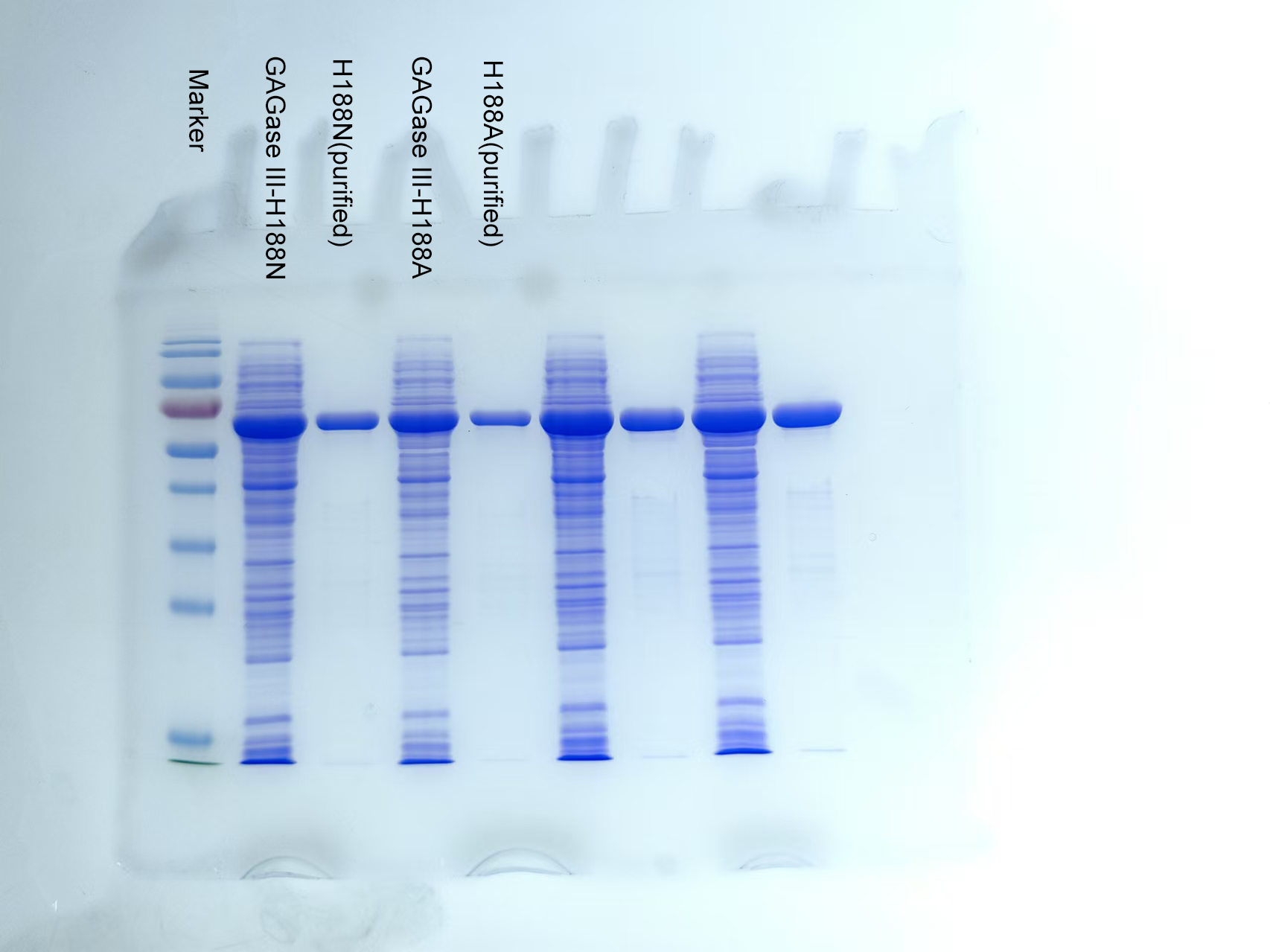

Supplement: Figure 4—figure supplement 2—source data 2. [file elife-102422-fig4-figsupp2-data2.zip › Figure 4-figure supplement 2 C-1.png]
