# Supplementary material for: Crystal structure and catalytic mechanism of PL35 family glycosaminoglycan lyases with an ultrabroad substrate spectrum
Source: eLife. 2025 May 19;13:RP102422. doi: 10.7554/eLife.102422 (PMC12088678; doi:10.7554/eLife.102422)
Supplement: Supplementary file 1. — (a) Sequence information of the identified glycosaminoglycan lyases (GAGases). (b) Inductively coupled plasma-mass spectrometry (ICP-MS) analysis of GAGase II and GAGase VII. (c) Strains and primers used in this study. [file elife-102422-supp1.docx]

*Supplementary* *data* for

**Crystal structure and catalytic mechanism of PL35 family glycosaminoglycan lyases with an ultrabroad substrate spectrum**

*Lin Wei^1,6^,* *Hai-Yan Cao^2,6^, Ruyi Zou^1^, Min Du^1^, Qingdong Zhang^3^,* *Danrong Lu^3^, Xiangyu Xu^1^,* *Yingying Xu^1^,* *Wenshuang Wang^1^, Xiu-Lan Chen^4,5^,* *Yu-Zhong Zhang^2,4,5^*,* *Fuchuan Li^1,5^**

^1^ National Glycoengineering Research Center and Shandong Key Laboratory of Carbohydrate Chemistry and Glycobiology, State Key Laboratory of Microbial Technology, Shandong University, Qingdao, China.

^2^ MOE Key Laboratory of Evolution and Marine Biodiversity, Frontiers Science Center for Deep Ocean Multispheres and Earth System & College of Marine Life Sciences, Ocean University of China, Qingdao, China.

^3^ School of Life Science and Technology, Weifang Medical University, Weifang, China.

^4^ Marine Biotechnology Research Center, State Key Laboratory of Microbial Technology, Shandong University, Qingdao, China.

^5^ Joint Research Center for Marine Microbial Science and Technology, Shandong University and Ocean University of China, Qingdao, China.

^6^ These authors contributed equally to this work.

* Corresponding authors. E-mail addresses: zhangyz@sdu.edu.cn (Y. Z.); fuchuanli@sdu.edu.cn (F. L.)

**Supplementary file 1a. Sequence information of the identified GAGases.**

|  | GenBank accession number | Originate | Sequence identity* (Query cover/Per. Ident) |
| --- | --- | --- | --- |
| GAGase II | SOD82962.1 | *Spirosoma fluviale* | 100%/100% |
| GAGase VII | EDV05210.1 | *Bacteroides intestinalis* DSM 17393 | 93%/44.7% |
| GAGase I | ADB38475.1 | *Spirosoma linguale* DSM 74 | 100%/81.21 |
| GAGase III | MBC7922758.1 | *Ferruginibacter* sp. | 98%/64.1% |
| GAGase IV | MPR36080.1 | *Prolixibacteraceae bacterium* | 99%/54.6% |
| GAGase V | HCY41582.1 | *Cytophagaceae bacterium* SJW1-29 | 99%/54.0% |
| GAGase VI | EKX96148.1 | *Prevotella saccharolytica* F0055 | 98%/49.1% |
| GAGase VIII | AVM53611.1 | *Bacteroides zoogleoformans* | 98%/42.7% |

*Sequence identity means the sequence similarity compared with GAGase II.

**Supplementary file 1b. Inductively coupled plasma-mass spectrometry (ICP-MS) analysis of GAGase II and GAGase VII.**

|  | Enzyme concentration | Ions  concentration | | | |
| --- | --- | --- | --- | --- | --- |
|  |  | Mn | Ca | Zn | Cu |
|  | (mg/ml) | (μg/L) | | | |
| Negative | - | 0.24 | 24.37 | 6.36 | 0.68 |
| GAGase II | 4.75 | 15.62 | 20.59 | 6.71 | 1.27 |
| GAGase VII | 2.70 | 21.58 | 24.345 | 9.84 | 1.25 |

**Supplementary file 1c. Strains and primers used in this study.**

|  | Description | | Source |
| --- | --- | --- | --- |
| Strain | |  |  |
| *Bacteroides intestinalis* DSM 17393  *E. coli* BL21(DE3) | | Intestinal microorganisms isolated from human faces  F^-^ *omp*T *hsd*S (rB^-^, mB^-^) *gal dcm* (DE3) | DSMZ |
|  |  |  | Vazyme Biotech. |
| Mutant primers | |  |  |
| GAGase II-Y246A-F | | 5’-TATAGCGCGTGGGGCTATGGCACGAGCTTTAA-3’ | Sangon Biotech |
| GAGase II-Y246A-R | | 5’-TAGCCCCACGCGCTATAGCCTTCCGGATACGC-3’ | . |
| GAGase II-H401A-F | | 5’-GCGGCGCACATGGATGTGGGCAGCTTTGTGAT-3’ |  |
| GAGase II-H401A-R | | 5’-ACATCCATGTGCGCCGCGCTCGTGCCCGGGCTGCC-3’ |  |
| GAGase II-N192A-F | | 5’-AACGCGTGGAACCAAGTGTGCAACGCGGGCAT-3’ |  |
| GAGase II-N192A-R | | 5’-ACTTGGTTCCACGCGTTGCTGCTGCGCAGCCA-3’ |  |
| GAGase III-Y243A-F | | 5’-TATGGCGCGTGGGGCTATGGCACGAGCTTTAA-3’ |  |
| GAGase III-Y243A-R | | 5’- TAGCCCCACGCGCCATAGCCTTCCGGATACGC-3’ |  |
| GAGase III-H398A-F | | 5’- GTGAACGCGGCGCACATGGATGTGGGCAGCTT |  |
| GAGase III-H398A-R | | 5’- ATGTGCGCCGCGTTCACGCTCGGGCT-3’ |  |
| GAGase III-N189A-F | | 5’-CATGCGTGGAACCAAGTGTGCAACG-3’ |  |
| GAGase III-N189A-R | | 5’-ACTTGGTTCCACGCATGGCTCGCTTTCAGCC-3’ |  |
| GAGase VII-Y241A-F | | 5’-TATTCCGCGTGGGGATATGGGACGACTTACAA-3’ |  |
| GAGase VII-Y241A-R | | 5’-TATCCCCACGCGGAATAGCCTTCGGCATACGC-3’ |  |
| GAGase VII-H397A-F | | 5’-ATCAGGAGCGACACATTTGGATGCCGGTTCTT-3’ |  |
| GAGase VII-H397A-R | | 5’-AATGTGTCGCTCCTGATTGTGCTGTCCCTCCT-3’ |  |
| GAGase VII-N187A-F | | 5’-TAATGCGTGGAATCAGGTATGTAATGGGGGAA-3’ |  |
| GAGase VII-N187A-R | | 5’-CCTGATTCCACGCATTATTTCGATAGAGCCAGCTATTATATT-3’ |  |
| GAGase II-R87A-F | | 5’-TATTCAGATTGGCGCGCGCCTGCTGGATAAAAGCC-3’ |  |
| GAGase II-R87A-R | | 5’-GCGCGCCAATCTGAATACGTTTCAGCGGTTCA-3’ |  |
| GAGase II-R88A-F | | 5’-CGCGCTGCTGGATAAAAGCCGCGAAGCGCTGC-3’ |  |
| GAGase II-R88A-R | | 5’-TTTATCCAGCAGCGCGCGGCCAATCTGAATACGTT-3’ |  |
| GAGase II-R94A-F | | 5’-ATAAAAGCGCGGAAGCGCTGCGCCGCATTTTT-3’ |  |
| GAGase II-R94A-R | | 5’-CGCTTCCGCGCTTTTATCCAGCAGGCGGCGGC-3’ |  |
| GAGase II-H136A-F | | 5’-ACCGCGTTTCTGGATGTGGCGGAAATGACCAT-3’ |  |
| GAGase II-H136A-R | | 5’-ACATCCAGAAACGCGGTCGGGTTCCAATCGCT-3’ |  |
| GAGase II-N191A-F | | 5’-GCGAACTGGAACCAAGTGTGCAACGCGGGCAT-3’ |  |
| GAGase II-N191A-R | | 5’-ACTTGGTTCCAGTTCGCGCTGCTGCGCAGCCAGCT-3’ |  |
| GAGase II-D233A-F | | 5’-GATGGGCGCGTATAAACCGGATGGCGCGTATC-3’ |  |
| GAGase II-D233A-R | | 5’-GTTTATACGCGCCCATCGGCAGCACCACCGCG-3’ |  |
| GAGase II-Y240A-F | | 5’-GGCGCCGGAAGGCTATAGCTATTGGGGCTATG-3’ |  |
| GAGase II-Y240A-R | | 5’-TATAGCCTTCCGGCGCCGCGCCATCCGGTTTATA-3’ |  |
| GAGase II-E242A-F | | 5’-GGCGGGCTATAGCTATTGGGGCTATGGCACGA-3’ |  |
| GAGase II-E242A-R | | 5’-AATAGCTATAGCCCGCCGGATACGCGCCATCCGG-3’ |  |
| GAGase II-Y249A-F | | 5’-ATAGCTATTGGGGCGCGGGCACGAGCTTTAACGTGATG-3’ |  |
| GAGase II-Y249A-R | | 5’-CGCGCCCCAATAGCTATAGCCTTCCGGATACG-3’ |  |
| GAGase II-D299A-F | | 5’-TAACTATAGCGCGAGCGGCCTGAGCGGCGAAC-3’ |  |
| GAGase II-D299A-R | | 5’-CGCTCGCGCTATAGTTATACGCGTTGCCGCTC-3’ |  |
| GAGase II-Q307A-F | | 5’-TGGCGCCGGCGATGTTTTGGTTTGCGAAAAAA-3’ |  |
| GAGase II-Q307A-R | | 5’-AAACATCGCCGGCGCCAGTTCGCCGCTCAGGCC-3’ |  |
| GAGase II-R340A-F | | 5’-AGAACCATCTGGCGAACCGCCTGCTGCCGGCG-3’ |  |
| GAGase II-R340A-R | | 5’-GTTCGCCAGATGGTTCTGCGGGTTGCTGTTCA-3’ |  |
| GAGase II-R342A-F | | 5’-ATCTGCGCAACGCCCTGCTGCCGGCGGCGCTG-3’ |  |
| GAGase II-R342A-R | | 5’-CAGGGCGTTGCGCAGATGGTTCTGCGGGTTGC-3’ |  |
| GAGase II-H403A-F | | 5’-CATGCGGCGATGGATGTGGGCAGCTTTGTGAT-3’ |  |
| GAGase II-H403A-R | | 5’-ACATCCATCGCCGCATGGCTCGTGCCCGGGCT-3’ |  |
| GAGase II-D405A-F | | 5’-ATGCGCACATGGCGGTGGGCAGCTTTGTGATGGA-3’ |  |
| GAGase II-D405A-R | | 5’-CACCGCCATGTGCGCATGGCTCGTGCCCGGGC-3’ |  |
| GAGase II-D421A-F | | 5’-ATGGCGTTTGGCATGCAAGAATATGAAAGCCT-3’ |  |
| GAGase II-D421A-R | | 5’-TGCATGCCAAACGCCATCGCCCAGCGCACGCC-3’ |  |
| GAGase II-M424A-F | | 5’-TTTTGGCGCGCAAGAATATGAAAGCCTGGAAAGC-3’ |  |
| GAGase II-M424A-R | | 5’-ATTCTTGCGCGCCAAAATCCATCGCCCAGCGC-3’ |  |
| GAGase II-Y427A-F | | 5’-TGCAAGAAGCGGAAAGCCTGGAAAGCAAAGGC-3’ |  |
| GAGase II-Y427A-R | | 5’-GCTTTCCGCTTCTTGCATGCCAAAATCCATCG-3’ |  |
| GAGase II-E428A-F | | 5’-AGAATATGCGAGCCTGGAAAGCAAAGGCGTGG-3’ |  |
| GAGase II-E428A-R | | 5’-CCAGGCTCGCATATTCTTGCATGCCAAAATCCA-3’ |  |
| GAGase II-E431A-F | | 5’-AAAGCCTGGCGAGCAAAGGCGTGGATCTGTGG-3’ |  |
| GAGase II-E431A-R | | 5’-TTTGCTCGCCAGGCTTTCATATTCTTGCATGC-3’ |  |
| GAGase II-W438A-F | | 5’-GGATCTGGCGAACATGAAACAGAACAGTCAGCGC-3’ |  |
| GAGase II-W438A-R | | 5’-TCATGTTCGCCAGATCCACGCCTTTGCTTTCC-3’ |  |
| GAGase II-M440A-F | | 5’-GTGGAACGCGAAACAGAACAGTCAGCGCTGGC-3’ |  |
| GAGase II-M440A-R | | 5’-TCTGTTTCGCGTTCCACAGATCCACGCCTTTG-3’ |  |
| GAGase II-R446A-F | | 5’-ACAGAACAGTCAGGCGTGGCAGATTCTGCGCTATAACA-3’ |  |
| GAGase II-R446A-R | | 5’-ACGCCTGACTGTTCTGTTTCATGTTCCACAGA-3’ |  |
| GAGase II-H457A-F | | 5’-ACTTTGCGGCGAACACCCTGAGCATTAACGATGA-3’ |  |
| GAGase II-H457A-R | | 5’-GTGTTCGCCGCAAAGTTGTTATAGCGCAGAAT-3’ |  |
| GAGase III-H188A-F | | GCGAACTGGAACCAAGTGTGCAACGCGGGCAT |  |
| GAGase III-H188A-R | | ACTTGGTTCCAGTTCGCGCTCGCTTTCAGCCAGTTGT |  |
| GAGase III-H188N-F | | AGCGAGCAATAACTGGAACCAAGTGTGCAACG |  |
| GAGase III-H188N-R | | TCCAGTTATTGCTCGCTTTCAGCCAGTTGTTA |  |
| GAGase II N191H-F | | AGCAGCCACAACTGGAACCAAGTGTGCAACGC |  |
| GAGase II N191H-R | | TTCCAGTTGTGGCTGCTGCGCAGCCAGCTGTT |  |
